# Combination of Telmisartan with Cisplatin Controls Oral Cancer Cachexia in Rats

**DOI:** 10.1155/2013/642848

**Published:** 2013-12-05

**Authors:** Bhoomika M. Patel, Deepak Damle

**Affiliations:** Institute of Pharmacy, Nirma University, Sarkhej-Gandhinagar Highway, Ahmedabad, Gujarat 382 481, India

## Abstract

The objective of the present investigation was to study the effect of combination of telmisartan with cisplatin in oral cancer cachexia induced by applying 0.5% 4-nitroquinoline-1-oxide (4-NQO) in propylene glycol to tongue, thrice a week for 8 weeks. From 8th to 22nd week, cisplatin (0.23 mg/kg, i.v.) was administered once in three weeks and telmisartan (5 mg/kg/day, p.o.) was administered daily. 4-NQO produced significant decrease in food intake, body weight, hyperglycemia, dyslipidemia, hypertension, and bradycardia, worsened hemodyanamics, increased cachexia markers like insulin, C-reactive protein, and interleukin-6, and increased tumor markers like lactate dehydrogenase and **γ**-glutamyl transferase.Treatment with combination of telmisartan with cisplatin produced significant increase in food intake and body weight and controlled hyperglycaemia and dyslipidemia, preserved hemodynamic function, and decreased the cachexia markers while cisplatin alone did not produce any increase in food intake and body weight. Further, the combination of telmisartan with cisplatin significantly reduced tumor marker levels. Combination of telmisartan with cisplatin prevented 4-NQO induced oxidative stress, hyperplasia and hyperkeratosis, premalignant dysplasia, and invasive squamous cell carcinoma in the tongue. Our data suggests that combination of telmisartan with cisplatin treatment is beneficial in controlling cancer cachexia. Telmisartan can be used as an add-on therapy with cisplatin or other traditional chemotherapeutic agents.

## 1. Introduction

Oral cancer is the sixth most common cancer reported globally with an annual incidence of over 300,000 cases, of which 62% arise in developing countries [1]. Cachexia is a well-recognized adverse effect of cancer, associated with reduced physical function, tolerance to anticancer therapy, and survival. Furthermore, weight loss in patients with cancer is rarely recognized, assessed, or managed actively [2]. A variety of metabolic and endocrine changes and activation of catabolic pathways account for some of the weight loss [3]. Thus, controlling cachexia becomes of utmost importance for improving the quality of life of cancer patients.

It has been reported that interleukin-6 (IL-6) is a trigger of the acute phase response [4]. Muscle atrophy has been described to occur in IL-6 transgenic mice [5], and inoculation of mice with Chinese hamster ovary tumor cells expressing IL-6 was found to be associated with body weight loss and hypophagia suggesting that IL-6 might be involved in the syndrome of cachexia [6]. Moreover, peroxisome proliferator-activated receptors-gamma (PPAR-*γ*) ligands, in addition to their role in diabetes and adipogenesis, are shown to inhibit tumor growth and progression in preclinical models of cancer, by modulating various cellular processes in cancer cells, stromal cells, and tumor microenvironment [7]. Thus, PPAR-*γ* agonists can be used for decreasing the energy expenditure in the patients suffering from cachexia and increasing the differentiation of adipocytes for producing an increase in weight of the cancer patients [8]. Additionally, angiotensin II directly induces muscle protein catabolism through the ubiquitin-proteasome proteolytic pathway contributing to progression of cachexia [8]. Thus, targeting IL-6, PPAR-*γ*, and Ang II appears to be an important strategy in control of cachexia.

Telmisartan is a specific AT_1_ receptor blocker with the strongest binding affinity to AT_1_ receptor among various ARBs [9] and is a partial agonist of peroxisome proliferator-activated receptor-*γ* (PPAR-*γ*) [10]. Telmisartan inhibits TNF-induced IL-6 expression at the transcriptional level through the activation of PPAR-*γ* [11]. It has also been reported that it prevents cardiovascular complications in various animal models of diabetes and hypertrophy [12–14]. In lieu of these facts, we hypothesized that adding telmisartan to cisplatin (which is widely used chemotherapeutic agent for oral cancer) may be beneficial in oral cancer cachexia using chemically induced model of oral cancer. Further, several models are available for cachexia like Yoshida AH-130 ascites hepatoma, Lewis lung carcinoma, C26 colon carcinoma, and so forth. However, there is no specific model of cachexia for oral cancer, and hence we used 4-nitroquinoline-1-oxide (4-NQO) induced oral cancer and evaluated cachexia in the same model.

## 2. Material and Methods

The protocol of the experiment was approved by Institutional Animal Ethics Committee as per the guidance of Committee for the Purpose of Control and Supervision of Experiments on Animals (CPCSEA), Ministry of Social Justice and Empowerment, Government of India (Protocol number: IPS/PCOL/MPH-11-12/005).

### 2.1. Animals

Wistar strain male albino rats of 6 weeks of age, weighing 250–350 g, were selected for the study and were maintained under well-controlled conditions of temperature (22 ± 2°C), humidity (55 ± 5%), and 12 h/12 h light-dark. Conventional laboratory diet (Pranav Agro Pvt. Ltd.) and UV-filtered water were provided *ad libitum*.

### 2.2. 4-NQO Induced Oral Cancer

The animals were randomly divided into 6 groups each containing six rats: Group I (CON) normal control, Group II (COC) control treated with cisplatin (0.23 mg/kg), Group III (COCT) control treated with combination of cisplatin (0.23 mg/kg) with telmisartan (5 mg/kg/day), Group IV (ORN) oral cancer control, Group V (ORC) oral cancer treated with cisplatin (0.23 mg/kg), and Group VI (ORCT) oral cancer treated with combination of cisplatin (0.23 mg/kg) with telmisartan (5 mg/kg/day). Groups I, II, and III received only propylene glycol thrice a week up to 8 weeks. In Groups IV, V, and VI, 0.5% 4-NQO in propylene glycol was applied to tongue by a paint brush of size number 4, thrice a week for 8 weeks. From 8–24 weeks, Groups II and V were given cisplatin (0.23 mg/kg) and Groups III and VI were given cisplatin (0.23 mg/kg) and telmisartan (5 mg/kg/day). The doses of cisplatin and telmisartan were decided on the basis of the clinical doses converted into animal doses using surface area and weight factors.

### 2.3. Blood Sample Collection and Serum Analysis

At the end of 24 weeks, blood samples were collected and allowed to clot and serum was separated by centrifuging, and they were analyzed for glucose, total cholesterol, VLDL-cholesterol, HDL-cholesterol, triglycerides, C-reactive protein (CRP), lactate dehydrogenase (LDH), and *γ*-glutamyl transferase (*γ*-GT), spectrophotometrically (Shimadzu UV-1601, Japan) using available biochemical diagnostic kits (Labcare Diagnostics Pvt. Ltd., India). Serum insulin was estimated by radioimmunoassay technique using gamma counter (Packard, USA). IL-6 was estimated from serum by enzyme immunoassay and ELISA reader (Biorad Laboratories Inc., USA).

### 2.4. Measurement of Hemodynamic Parameters

Invasive blood pressure monitoring technique was adopted as described elsewhere [15, 16]. The animals were anaesthetized by urethane (1.5 mg/kg, i.p) and diazepam (4 mg/kg, i.m). The carotid artery behind the trachea was exposed and cannulated for the measurement of hemodynamic parameters using a transducer (BP 100) and Labscribe system (IWORX, New Hampshire, USA). The hemodynamic parameters observed were systolic blood pressure (SBP), diastolic blood pressure (DBP), heart rate, rate of pressure development (*dp/dt*_max_), and rate of pressure decay (*dp/dt*_min_). All the data were analyzed using Labscribe software (Version 118).

### 2.5. Measurement of Tissue Parameters

At the end of the study, animals were sacrificed, and tongue was excised and was homogenized in Tris-HCl buffer (0.01 M, pH. 7.4) using a REMI homogenizer (REMI Motor, Bombay, India) to generate a 10% homogenate. The homogenate was subjected to estimation of malondialdehyde (MDA) [17], reduced glutathione (GSH) [18], and superoxide dismutase (SOD) levels [19]. Briefly, for estimation of MDA levels, 0.2 mL of homogenate was taken and mixed with 0.2 mL of sodium lauryl sulphate, 1.5 mL acetic acid in HCl, 1.5 mL thiobarbituric acid, and 0.6 mL distilled water. The mixture was heated for 45 min in water bath at 95°C and was allowed to cool and then was mixed with 5 mL mixture of *n*-butanol : pyridine. The absorbance was read against blank at 532 nm. For the measurement of GSH levels, 0.2 mL of supernatant was taken and mixed with 1 mL of 10% trichloroacetic acid. The mixture was kept in ice bath for 30 min and then centrifuged for 10 min at 4°C at 3000 RPM. Form this, 0.5 mL of supernatant was taken and mixed with 2 mL disodium hydrogen phosphate and 0.25 mL dithiobis nitrobenzoic acid. The absorbance was read against blank at 412 nm. SOD levels were measured by taking 0.2 mL of supernatant and mixing with 0.1 mL EDTA, 0.5 mL carbonate buffer, and 1 mL epinephrine. The absorbance was read against blank at an interval of 30 sec. for 3 min. at 480 nm. The tongue was also subjected to histopathological studies for hematoxylin and eosin (HE) staining. The sections were observed and desired areas were photographed in an Olympus photomicroscope under 40x magnification.

### 2.6. Statistical Analysis

Results are presented as mean ± SEM. Statistical differences between the means of the various groups were evaluated using one-way analysis of variance (ANOVA) followed by Tukey's test. Data were considered statistically significant at *P* value < 0.05.

## 3. Results

### 3.1. Food Intake and Body Weight

**Figure 1:**
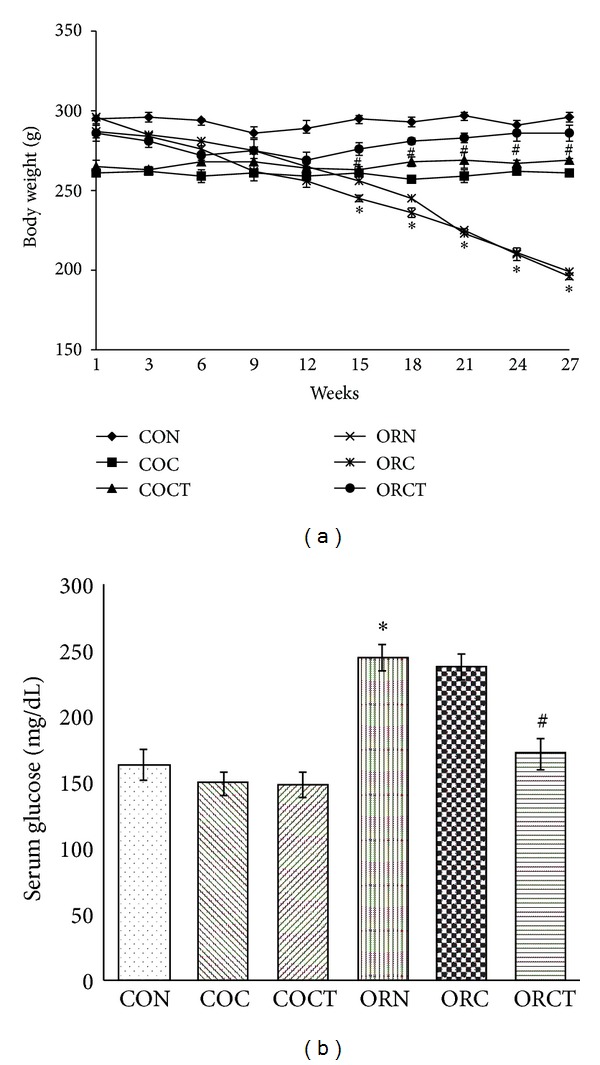
Effect of cisplatin and combination of cisplatin with telmisartan on change in (a) body weight and (b) serum glucose levels. Each point represents mean ± SEM of 6 experiments from 0 to 27 weeks. Each bar represents mean ± SEM of 6 experiments analyzed at the end of 24 weeks. *Significantly different from control group (*P* < 0.05), ^#^significantly different from cancer control group (*P* < 0.05), CON: normal control, COC: control treated with cisplatin (0.23 mg/kg), COCT: control treated with cisplatin (0.23 mg/kg) + telmisartan (5 mg/kg/day), ORN: oral cancer control, ORC: oral cancer treated with cisplatin (0.23 mg/kg), and ORCT: oral cancer treated with cisplatin (0.23 mg/kg) + telmisartan (5 mg/kg/day).

4-Nitroquinoline-N-oxide (4-NQO) produced a significant (*P* < 0.05) decrease in body weight (Figure 1(a)) in cancer control group as compared to normal control group. Treatment with cisplatin alone did not show any significant (*P* < 0.05) increase in body weight. However, treatment with combination of cisplatin with telmisartan showed significant (*P* < 0.05) increase in body weight (Figure 1(a)) in cancer treated rats as compared to cancer control rats. There was no significant change in food intake of the cancer control and cancer treated rats (Table 1).

### 3.2. Serum Glucose Levels and Lipid Profile

4-NQO treated rats exhibited significantly (*P* < 0.05) increased serum glucose (Figure 1(b)) level as compared to normal control group. Treatment with cisplatin alone did not show any significant (*P* < 0.05) change in serum glucose; however, treatment with combination of cisplatin with telmisartan showed significant (*P* < 0.05) decrease in serum glucose levels in cancer treated rats as compared to cancer control rats (Figure 1(b)).

Cancer control rats were found to exhibit significant (*P* < 0.05) decreased levels of serum total cholesterol, triglyceride and HDL, and VLDL as compared to normal control group. Treatment with cisplatin alone did not show any significant (*P* < 0.05) change in serum cholesterol, triglyceride and HDL, and VLDL levels; however, treatment with combination of cisplatin with telmisartan produced significant (*P* < 0.05) increase in serum cholesterol, triglyceride and HDL, and VLDL levels in cancer treated rats as compared to cancer control rats (Table 1).

### 3.3. Hemodynamic Parameters

**Figure 2:**
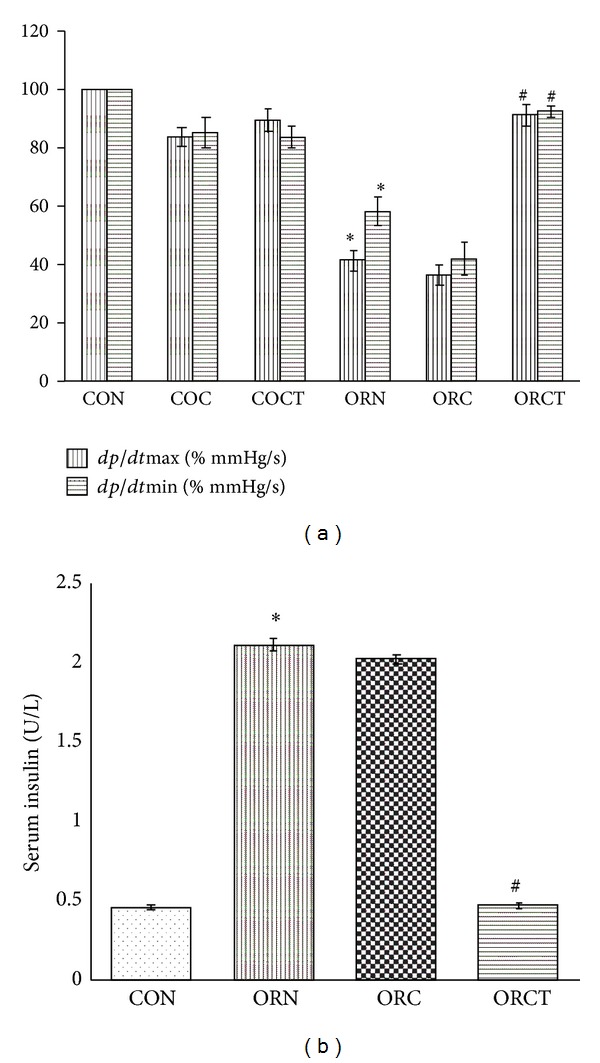
Effect of cisplatin and combination of cisplatin with telmisartan on change in (a) rate of pressure development and decay and (b) serum insulin levels. Each bar represents mean ± SEM of 6 experiments analyzed at the end of 24 weeks.*Significantly different from control group (*P* < 0.05), ^#^significantly different from cancer control group (*P* < 0.05), CON: normal control, COC: control treated with cisplatin (0.23 mg/kg), COCT: control treated with cisplatin (0.23 mg/kg) + telmisartan (5 mg/kg/day), ORN: oral cancer control, ORC: oral cancer treated with cisplatin (0.23 mg/kg), and ORCT: oral cancer treated with cisplatin (0.23 mg/kg) + telmisartan (5 mg/kg/day).

4-NQO treated rat exhibited significant (*P* < 0.05) increase in blood pressure (Table 2) and significant (*P* < 0.05) decrease in heart rate (Table 2) and rate of pressure development and decay (Figure 2(a)) as compared to control rats. Treatment with cisplatin alone did not show any significant (*P* < 0.05) change in blood pressure, heart rate, and rate of pressure development and decay; however, treatment with combination of cisplatin with telmisartan showed significant (*P* < 0.05) decrease in blood pressure (Table 2) and significant (*P* < 0.05) increase in heart rate (Table 2) and rate of pressure development and decay (Figure 2(a)) in cancer treated rats as compared to cancer control rats.

### 3.4. Cachexia Markers

Cancer control rats were found to exhibit significant (*P* < 0.05) increased serum insulin levels as compared to normal control group. Treatment with cisplatin alone did not show any significant (*P* < 0.05) change in elevated serum insulin levels; however, treatment with combination of cisplatin with telmisartan produced significant (*P* < 0.05) decrease in elevated serum insulin levels in cancer treated rats (Figure 2(b)).

**Figure 3:**
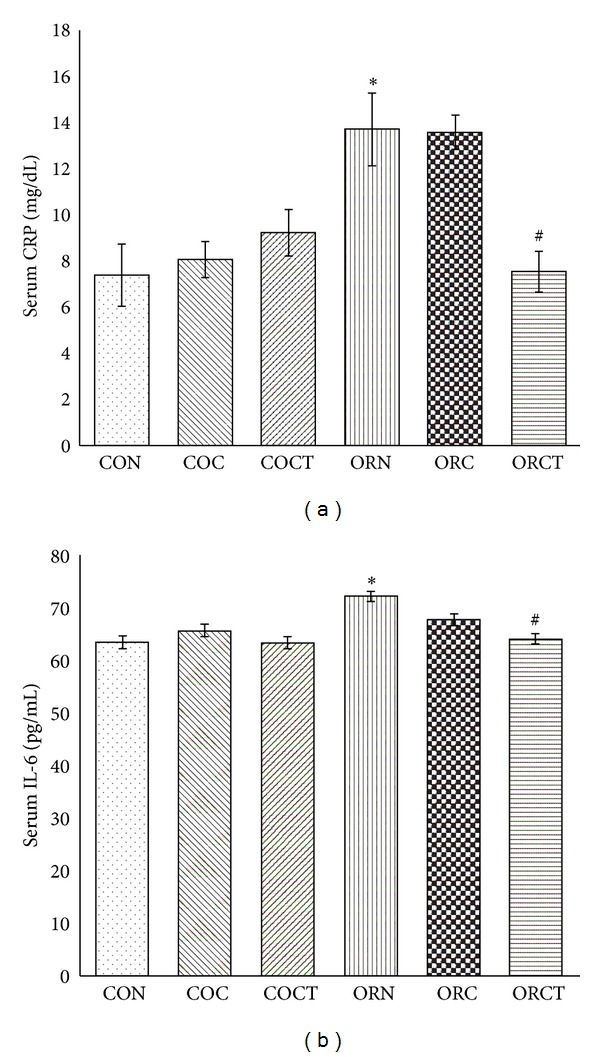
Effect of cisplatin and combination of cisplatin with telmisartan on change in (a) serum C-reactive protein levels and (b) serum interleukin-6 levels. Each bar represents mean ± SEM of 6 experiments analyzed at the end of 24 weeks.*Significantly different from control group (*P* < 0.05), ^#^significantly different from cancer control group (*P* < 0.05), CON: normal control, COC: control treated with cisplatin (0.23 mg/kg), COCT: control treated with cisplatin (0.23 mg/kg) + telmisartan (5 mg/kg/day), ORN: oral cancer control, ORC: oral cancer treated with cisplatin (0.23 mg/kg), and ORCT: oral cancer treated with cisplatin (0.23 mg/kg) + telmisartan (5 mg/kg/day).

4-NQO treated rats exhibited significantly (*P* < 0.05) increased CRP level as compared to normal control group. Treatment with cisplatin alone did not show any significant (*P* < 0.05) change in CRP levels; however, treatment with combination of cisplatin with telmisartan showed significant (*P* < 0.05) decrease in CRP level in cancer treated rats as compared to cancer control rats (Figure 3(a)).

4-NQO treated rats exhibited significantly (*P* < 0.05) increased IL-6 level as compared to normal control group. Treatment with cisplatin alone did not show any significant (*P* < 0.05) change in IL-6 levels; however, treatment with combination of cisplatin with telmisartan showed significant (*P* < 0.05) decrease in IL-6 level in cancer treated rats as compared to cancer control rats (Figure 3(b)).

### 3.5. Tumor Markers

4-NQO treated rats exhibited significantly (*P* < 0.05) increased serum LDH levels as compared to normal control rats. Treatment with cisplatin alone showed significant (*P* < 0.05) decrease in LDH levels in cancer treated rats as compared to cancer control rats. However, combination of cisplatin with telmisartan produced a decrease in LDH levels in cancer treated rats which was significantly (*P* < 0.05) lower than that of cisplatin treatment alone (Table 3).

4-NQO treated rat exhibited significantly (*P* < 0.05) increased serum *γ*-GT levels as compared to normal control group. Treatment with cisplatin alone showed significant (*P* < 0.05) decrease in *γ*-GT levels in cancer treated rats as compared to cancer control rats. However, combination of cisplatin with telmisartan also produced a decrease in *γ*-GT levels in cancer treated rats; however, this decrease was significantly (*P* < 0.05) different than that of cisplatin treatment alone.

4-NQO produced a significant (*P* < 0.05) increase in levels of tongue tissue MDA and significant (*P* < 0.05) decrease in tongue tissue SOD and GSH levels as compared to control rats. Treatment with cisplatin alone showed significant (*P* < 0.05) decrease in tongue tissue MDA and significant (*P* < 0.05) decrease in tongue tissue SOD and GSH levels. However, combination of cisplatin with telmisartan produced a decrease in tongue tissue MDA and significant (*P* < 0.05) decrease in tongue tissue SOD and GSH levels which were significantly (*P* < 0.05) different than those of cisplatin treatment alone (Table 3).

### 3.6. Histopathological Studies

**Figure 4:**
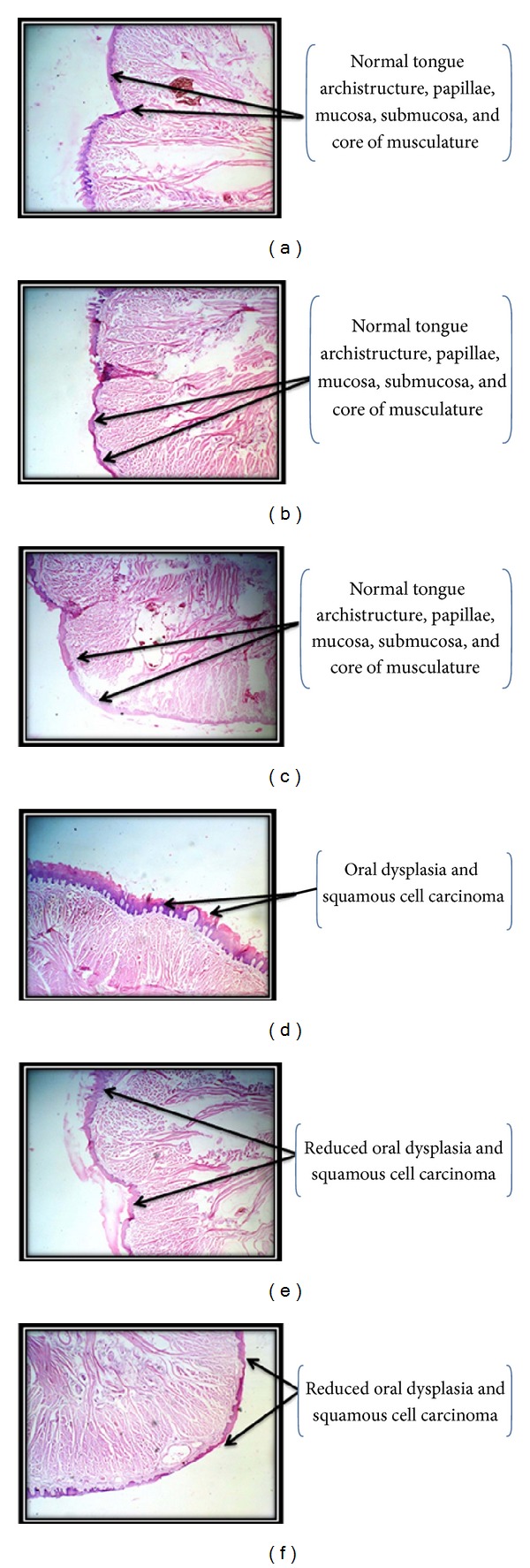
Representative figure of histology of tongue tissue analyzed at the end of 24 weeks from (a) normal control, (b) control treated with cisplatin, (c) control treated with combination of cisplatin with telmisartan, (d) oral cancer control, (e) oral cancer treated with cisplatin and (f) oral cancer treated with combination of cisplatin with telmisartan. Magnification ×40.

Histopathological analysis of Wistar rats tongue from normal control group, control treated with cisplatin, and from that with combination of cisplatin with telmisartan (Figures 4(a), 4(b), and 4(c)) showed normal tongue archistructure, papillae, mucosa, submucosa, and core of musculature. No sign of inflammation or tissue damage and erosions/hyperplasic lesions were seen. Oral cancer control group animals' tongue histopathology showed moderate and severe oral dysplasia and squamous cell carcinoma (Figure 4(d)). The tumors spread into the submucosa and the underlying muscle layer, forming small nests with typical keratin pearl formation, which was significantly reduced in group treated with cisplatin (Figure 4(e)) and combination of cisplatin with telmisartan (Figure 4(f)).

## 4. Discussion

Cancer cachexia is characterized by chronic wasting syndrome and anorexia, involving loss of both adipose tissue and lean body mass [20]. In the present study, we have studied the effect of addition of telmisartan to conventionally used chemotherapeutic agent cisplatin to investigate the effect of telmisartan on cancer cachexia.

In the present study, 4-NQO produced reduced food intake and loss of body weight among the cancer control animals which were controlled by treatment with combination of telmisartan with cisplatin and not with cisplatin alone. It has been reported that NF-*κ*B and C/EBP DNA binding activity are responsible for TNF-*α* induced IL-6 expression via PPAR-*γ*, and inhibition of PPAR-*γ* might be the reason for decreased levels of TNF-*α* induced IL-6 expression which are mediators of anorexia [21]. Thus, telmisartan being a partial agonist of PPAR-*γ* justifies the significant increase in food intake which accounts for the increased body weight.

Cancer patients exhibit higher serum glucose levels which might be due to the decreased glucose uptake caused by suppression of tyrosine phosphokinase via increased tissue TNF-*α* [22]. In the present investigation, 4-NQO produced significantly higher serum glucose levels among the cancer control animals which were reversed after treatment with combination of telmisartan with cisplatin and not with cisplatin alone. It has been reported that PPAR-*γ* agonist decreases TNF-*α* in skeletal muscle and increases insulin sensitivity [23]. Thus, telmisartan being a partial agonist of PPAR-*γ* justifies the significant decreased in serum glucose levels. Reduction in glucose levels by telmisartan treatment suggests improvement in glucose utilization by host tissue.

It has been reported that there occurs an inverse association between blood cholesterol levels and risk of cancer [24] and low HDL is a predictor of cancer [25]. In the present investigation, combination of telmisartan with cisplatin controlled the 4-NQO induced dyslipidemia while cisplatin alone did not produce any improvement. It has been reported that angiotensin II stimulates NADPH oxidase activity via the AT_1_ receptor to induce oxidative stress [26] which may further increase utilization of lipid. Telmisartan being an antagonist at AT_1_ receptor might prevent angiotensin II mediated oxidative stress. Moreover, it has also been reported that IL-6 upregulates expression of AT_1_ receptor and overexpression of AT_1_ receptors leads to increased oxidative stress [27]. As discussed earlier, telmisartan through PPAR-*γ* agonist activity decreases IL-6 expression and thus decreases IL-6 mediated upregulation of AT_1_ receptor which further supports improvement in the lipid profile by telmisartan treatment.

Increase in activity of renin-angiotensin system has been reported in cancer conditions [28]. In the present study, combination of telmisartan with cisplatin reduced 4-NQO induced elevated blood pressure while cisplatin alone did not. Telmisartan is a potent antihypertensive agent acting through AT_1_ antagonism, and hence reduction in blood pressure is justified among treated animals.

Elevated energy expenditure is related to the increased heart rate in cancer patients in a significantly higher proportion than that in controls [29]. In our study, combination of telmisartan with cisplatin reduced 4-NQO induced elevated heart rate while cisplatin alone did not. Ang II exerts an inhibitory influence on the baroreceptor reflex control of heart rate, which is mediated by the AT_1_ receptor [30]. Because telmisartan is an AT_1_ receptor antagonist, it is possible that it may attenuate the Ang II-mediated inhibition of the baroreceptor reflex.

Increased cytokine levels in cachexia stimulate NF-*κ*B signaling, which then mediates muscle loss and produces significant cardiac dysfunction [31]. The left ventricular dysfunction has been associated with a decrease in rate of pressure of pressure development and decay [32, 33]. In the present study, combination of telmisartan with cisplatin improved the hemodynamic functions while cisplatin alone did not. Therefore, the results of the hemodynamic study indicate the beneficial role of telmisartan in cardiovascular dysfunction.

Insulin, CRP, and IL-6 are biomarkers of cachexia [34]. Studies suggest that insulin resistance can result in increased protein degradation and the wasting of skeletal muscle [35]. Cancer patients have higher insulin levels due to insulin resistance in peripheral tissues [22]. In the present investigation, 4-NQO produced significantly higher serum insulin levels among the cancer control animals which were reversed after treatment with combination of telmisartan with cisplatin and not with cisplatin alone. PPAR-*γ* increases insulin sensitivity via several mechanisms like vasodilatation [36], inhibition of the impairment of insulin signaling induced by angiotensin II [37], and increase in the ratio of insulin-sensitive type 1 fiber in muscle fiber composition [38]. Thus, telmisartan being a partial agonist of PPAR-*γ* justifies the significant decreased in serum insulin levels, suggesting that addition of telmisartan to cisplatin treatment may prevent insulin resistance mediated protein degradation and skeletal muscle wasting.

C-reactive protein (CRP) is a nonspecific but sensitive marker of inflammation [39] and its release is mediated by IL-6 [40]. It is positively correlated with weight loss, anorexia-cachexia syndrome, extent of disease, and recurrence in advanced cancer [40]. In the present investigation, combination of telmisartan with cisplatin reduced 4-NQO induced increase in CRP levels while cisplatin alone did not. It has been reported that IL-6, IL-1, and TNF-*α* induce synthesis of CRP in hepatocytes [39]. As discussed earlier, telmisartan through PPAR-*γ* agonist attenuates IL-6 expression, and it might decrease IL-6 mediated upregulation of synthesis of CRP. Thus, telmisartan treatment justifies the significant decrease in CRP levels.

IL-6 is a trigger of the acute phase response [4]. Elevated levels of IL-6 are found in weight-losing cancer patients [41], and antibodies against IL-6 will suppress the development of cachexia in animals to some extent [42]. In the present study, there was significant increase in IL-6 levels in NQO treated rats. The combination of telmisartan with cisplatin reduced the IL-6 levels while cisplatin treatment did not. Telmisartan through PPAR-*γ* agonist activity decreases IL-6 expression [21]. Thus, the reduction in IL-6 levels by addition of telmisartan to cisplatin treatment further confirms the mechanism of prevention of cachexia by telmisartan.

From the ongoing discussion, it appears that telmisartan is beneficial in controlling cachexia as evident from the improvement in cachexia markers namely, insulin, CRP, and IL-6. Hence, we thought that it is worthwhile to explore the effects of telmisartan on tumor progression. In lieu of this, we estimated various markers like lactate dehydrogenase, *γ*-glutamyl transpeptidase, oxidative stress and histopathological study.

The main cause of weight loss in cachexia is massive hepatic gluconeogenesis caused primarily by the switch from aerobic to anaerobic dissimilation in which acetate dehydrogenase (LDH) catalyzes the conversion of lactate to pyruvate and vice versa [43, 44]. In the present investigation, 4-NQO produced significantly increased levels of serum LDH. Treatment with cisplatin significantly decreased LDH levels, but combination of cisplatin with telmisartan produced a decrease in LDH which was lower than that of cisplatin treatment alone suggesting a synergistic action of the combination. It has been reported that increased hepatic glucose production occurs due to a lack of inhibition of gluconeogenesis by insulin resistance [45]. As telmisartan is known to increase sensitivity of insulin, it justifies the significant decrease in LDH levels.

Studies have suggested that *γ*-glutamyl transpeptidase (GGT) is elevated in various primary and secondary malignancies including brain tumours, gastrointestinal tract, oral cavity, and so forth [46]. In the present investigation, 4-NQO produced significantly higher levels of GGT. Treatment with cisplatin significantly decreased GGT levels, but combination of cisplatin with telmisartan produced a decrease in GGT which was lower than that of cisplatin treatment again suggesting a synergistic action of the combination. It has been reported that the glutathione and GGT exhibit reciprocal relationships [47]. Telmisartan can prevent the activation of NF-*κ*B signaling pathway which promotes the transcription of NADPH oxidase and iNOS genes, and thus this may be the probable mechanism behind the decrease in GGT levels. Thus, though cisplatin is a potent chemotherapeutic agent, combination of cisplatin with telmisartan showed better improvement as far as the tumor markers are concerned.

GSH plays an important role in scavenging reactive oxygen species protecting the cell against cytotoxic and carcinogenic chemicals [48]. Overexpression of MnSOD in numerous transformed cell lines leads to reversion of the tumorigenicity *in vivo* or of the malignant phenotype *in vitro* [49, 50]. In the present study, we found significantly lower levels of GSH and SOD in cancer control rats. Treatment with cisplatin significantly increased GSH and SOD levels, but combination of cisplatin with telmisartan produced more increase than that of cisplatin treatment alone. It has been reported that the glutathione and GGT exhibit reciprocal relationships [47]. As mentioned earlier telmisartan decreases GGT levels which justifies the increase in glutathione levels.

Oxidative stress is as an imbalance between the production and scavenging of reactive oxygen species (ROS) with measurable increases in ROS in the cells and in the extracellular milieu [51]. In the present investigation, 4-NQO produced significant increase in MDA levels as compared to normal control animals. Treatment with cisplatin significantly decreased MDA levels, but combination of cisplatin with telmisartan produced a decrease in MDA which was lower than that of cisplatin treatment. It has been reported that angiotensin II stimulates NADPH oxidase activity via the AT_1_ receptor to produce the superoxide anion, hydrogen peroxide, and hydroxyl radicals [26]. It has also been reported that IL-6 upregulates expression of AT_1_ receptor, leading to increased oxidative stress [27]. Telmisartan being an AT_1_ receptor antagonist inhibits AT_1_ receptor mediated generation of free radicals and thereby controls oxidative stress. This further supports the contention that addition of telmisartan to cisplatin treatment may serve in controlling the tumor progression.

4-NQO produces histopathological changes in tongue mucosa from normal epithelium, hyperplasia and hyperkeratosis, premalignant dysplasia, and carcinoma in situ to invasive squamous cell carcinoma similar to those in humans [52]. In the present investigation, there were significant hyperplasia, hyperkeratosis, epithelial dysplasia, and squamous cell carcinoma of well-differentiated type among the oral cancer control animals which was reduced significantly upon treatment with cisplatin. However, combination of telmisartan with cisplatin showed better improvement in histology of tongue.

## 5. Conclusions

In conclusion, our data suggests that combination of telmisartan with cisplatin is beneficial in controlling cancer cachexia as depicted by decreased levels of cachexia and tumor markers. This effect of telmisartan is mediated through inhibition of IL-6 levels. Thus, telmisartan can be used as an add-on therapy with cisplatin or other traditional chemotherapeutic agents for treatment of oral cancer and control cachexia, thereby increasing the quality of life of cancer patients.

## Figures and Tables

**Table 1:** Effect of cisplatin and combination of cisplatin with telmisartan on food intake and serum lipid profile.

Parameters	CON	COC	COCT	ORN	ORC	ORCT
Food intake (gm/group/day)	143.73±9.24	141.11±9.21	144.53±10.57	125.37±13.15	132.78±7.63	140.56±8.70
Serum cholesterol (mg/dL)	82.13±5.56	78.51±4.97	78.31±2.91	45.84±1.60*	67.43±4.75	74.88±8.14^#^
Serum VLDL (mg/dL)	26.88±1.51	28.66±0.64	31.78±2.28	17.15±0.78*	107.73±2.33	27.52±1.42^#^
Serum triglyceride (mg/dL)	134.41±7.56	143.34±3.22	158.93±11.42	85.77±3.93*	107.73±14.12	130.54±6.20^#^
Serum HDL (mg/dL)	81.93±5.86	72.87±3.64	90.34±6.21	40.33±8.50*	56.20±6.52	78.78±4.11*

*Significantly different from control group (*P* < 0.05).

^#^Significantly different from cancer control group (*P* < 0.05).

CON: normal control.

COC: control treated with cisplatin (0.23 mg/kg).

COCT: control treated with cisplatin (0.23 mg/kg) + telmisartan (5 mg/kg/day).

ORN: oral cancer control.

ORC: oral cancer treated with cisplatin (0.23 mg/kg).

ORCT: oral cancer treated with cisplatin (0.23 mg/kg) + telmisartan (5 mg/kg/day).

**Table 2:** Effect of cisplatin and combination of cisplatin with telmisartan on hemodynamic parameters.

Parameters	CON	COC	COCT	ORN	ORC	ORCT
Systolic blood pressure (mmHg)	154.15±10.19	176.84±6.13	140.69±0.032	177.74±6.52	164.03±2.77	139.48±5.94^#^
Diastolic blood pressure (mmHg)	136.95±7.66	145.16±1.81	123.95±1.14	147.37±2.81*	135.40±1.33	124.41±1.46^#^
Heart rate (beats per minute)	251.95±7.35	247.68±1.93	238.50±3.01	254.89±5.25	227.54±5.60	223.71±3.08

*Significantly different from control group (*P* < 0.05).

^#^Significantly different from cancer control group (*P* < 0.05).

CON: normal control.

COC: control treated with cisplatin (0.23 mg/kg).

COCT: control treated with cisplatin (0.23 mg/kg) + telmisartan (5 mg/kg/day).

ORN: oral cancer control.

ORC: oral cancer treated with cisplatin (0.23 mg/kg).

ORCT: oral cancer treated with cisplatin (0.23 mg/kg) + telmisartan (5 mg/kg/day).

**Table 3:** Effect of cisplatin and combination of cisplatin with telmisartan on tumor markers.

Parameters	CON	COC	COCT	ORN	ORC	ORCT
Serum lactate dehydrogenase (U/l)	835.54±26.86	763.02±18.19	687.76±20.88	996.71±21.19*	964.62±25.98	832.24±30.05^#^
Serum *γ*-glutamyl transferase (U/l)	74.62±3.752	75.85±2.053	67.05±7.211	104.003±1.672*	103.32±3.037	78.85±2.995^#^
Tissue reduced glutathione (*μ*g/mg protein)	0.115±0.005	0.121±0.001	0.132±0.003	0.088±0.003*	0.104±0.007	0.114±0.001^#^
Tissue super oxide dismutase (units/mg protein)	3.59±0.17	2.70±0.168	2.72±0.166	4.47±0.094*	3.80±0.229	3.43±0.109^#^
Tissue malondialdehyde (nmoles/mg protein)	0.084±0.009	0.090±0.004	0.138±0.028	0.151±0.007*	0.097±0.007	0.096±0.004^#^

*Significantly different from control group (*P* < 0.05).

^#^Significantly different from cancer control group (*P* < 0.05).

CON: normal control.

COC: control treated with cisplatin (0.23 mg/kg).

COCT: control treated with cisplatin (0.23 mg/kg) + telmisartan (5 mg/kg/day).

ORN: oral cancer control.

ORC: oral cancer treated with cisplatin (0.23 mg/kg).

ORCT: oral cancer treated with cisplatin (0.23 mg/kg) + telmisartan (5 mg/kg/day).
